# Safety and Efficacy of Zynrelef^®^ in Combination with a Single Unilateral or Bilateral Nerve Block Performed Prior to Surgery

**Published:** 2023-10-05

**Authors:** Shiv Goel, Charles Luke, Matthew Holtzman, Benjamin Davies, Michael O’Malley, Dani Lavage, Carley Siedlecki, Jacques E Chelly

**Affiliations:** 1 Department of Anesthesiology and Perioperative Medicine, University of Pittsburgh Medical Center, Pennsylvania, USA; 2 Department of Surgery, UPMC Shadyside Hospital, Pennsylvania, USA; 3 Department of Urology, UPMC Shadyside Hospital, Pennsylvania, USA; 4 Department of Orthopedic Surgery, UPMC Shadyside Hospital, Pennsylvania, USA

**Keywords:** Pain, Zynrelef^®^, Nerve blocks, Ambulatory surgery, Open surgery, Opioids, NSAIDs

## Abstract

**Purpose:**

The FDA recently approved Zynrelef^®^ (A viscous solution of extended release of bupivacaine and meloxicam) to be applied at closure and providing postoperative analgesia for 72 hrs. Although the FDA didn’t restrict the use of nerve blocks in combination with this formulation, the safety and efficacy of such a combination has yet to be documented. This quality improvement study investigated this combination within the FDA-approved indications.

**Methods:**

Selected surgeons at two hospitals were chosen to use Zynrelef^®^. According to the standard of care, surgeons were also allowed to request single nerve blocks before surgery. The type of nerve blocks (unilateral or bilateral) performed included quadratus lumborum and paravertebral blocks for abdominal surgery, and adductor canal block for total knee replacement. Each block was performed with 20 mL of 0.375% bupivacaine (n=129) or 0.5% of ropivacaine (n=30). Pain scores, opioid consumption, and prescription refill requests at discharge were recorded. Patients discharged on the same day of surgery were separated into two groups-those who received single nerve blocks plus an Zynrelef^®^ (group 1) *vs.* those receiving Zynrelef^®^ only (group 2) and was analyzed using an un-paired t-test.

**Results:**

A total of 184 patients received Zynrelef^®^, including 25 patients who didn’t receive blocks, 44 who received unilateral blocks and 114 who received bilateral blocks. No symptoms suggestive of Local Anesthetic Toxicity (LAST) were observed. The use of the combination was associated with a 50% reduction in the number of patients filling their opioid prescription.

**Conclusion:**

This study provides evidence that the combination of a single unilateral or bilateral nerve block with Zynrelef^®^ is safe.

## Introduction

Despite the current opioid epidemic and the risk of opioid use disorders associated with persistent opioid use in surgical patients, opioids remain the default treatment for perioperative pain management, even for cases treated using a multimodal approach [1]. This leads to opioid prescriptions at the time of discharge, and in many cases, requests from patients for opioid refills, leading to persistent opioid use [2].

Zynrelef^®^ (HTX-011; Zynrelef^®^) is a viscous solution containing an extended-release formulation of bupivacaine (29.25 mg/ml) and meloxicam (0.88 mg/ml). It has been approved by the FDA to provide 72 hours of postoperative analgesia when applied into the surgical site at the time of closure following abdominal, foot, and primary knee joint replacement surgeries. This approval is based on studies conducted on patients undergoing open herniorrhaphy, Total Knee Arthroplasty (TKA), and bunionectomy. In these studies, 51.2% to 77% of patients in Zynrelef^®^ group did not receive opioids, and 93% of patients were discharged without a prescription for opioids [3–5]. In the TKA study, 56 patients randomized to Zynrelef^®^ group also received a local injection of 50 mg ropivacaine. Such a combination was found safe [5].

Although the bupivacaine plasma levels generated by Zynrelef^®^ are six to eight times lower than the toxic level of bupivacaine and the FDA has not placed any restrictions on the use of blocks in patients receiving Zynrelef^®^, the safety and efficacy of the combination of Zynrelef^®^ with single nerve blocks performed prior to surgery remain undocumented [6–8]. The objective of this quality improvement study was to document the efficacy of using a single nerve block in conjunction with Zynrelef^®^ for the FDA-approved indications. A safety committee was established to monitor the use of Zynrelef^®^ and review protocol deviations and any cases of local anesthetic toxicity associated with the use of Zynrelef^®^ alone or in combination with single nerve blocks.

## Materials and Methods

This study was conducted at two community hospitals from July 1, 2021 to March 3, 2023. The study was reviewed and approved by The University of Pittsburgh quality improvement committee (QI^#^ 3803). The approval was based on the committee assessment that the proposed project didn’t meet the federal definition of research according to 45 CFR 46.102(l) and therefore did not require additional IRB oversight. The study was complied with the Declaration of Helsinki. Following the FDA-approved indications, it was decided that patients qualifying for administration of Zynrelef^®^ would include patients undergoing same day admission for an abdominal surgery (small and medium incisions), TKA, or foot and ankle surgery. A selected number of surgeons were allowed to use Zynrelef^®^. These surgeons were also allowed to consult the acute pain service for single nerve blocks performed preoperatively. After consenting to receive Zynrelef^®^ prior to surgery, a specific bracelet was placed on their wrist prior to the transfer to the operating room.

A monitoring committee comprising the chief of anesthesiology, a member of the acute pain service, the director of the operating room, the director of the same day surgery and recovery room, the director of pharmacy, operating room pharmacists, anesthesiologists, nurses, and a surgeon was established with the mission to monitor the use of Zynrelef^®^ and establish a protocol defining the conditions by which Zynrelef^®^ should be used. The protocol included: 1) Exclusion of both continuous nerve blocks or intravenous infusions of lidocaine; 2) request that surgeons interested in using Zynrelef^®^ make a formal request at least 24 hours in advance to allow the operating room pharmacy to prepare to deliver Zynrelef^®^ and post on the nerve block schedule to prevent the placement of nerve block catheters and orders for postoperative continuous infusion of either perineural or IV local anesthetics; 3) placement of Zynrelef^®^ bracelet by the nurse in the same Day of Admission Surgery unit (DAS); and 4) request that the certified registered nurse anesthetist document the use of Zynrelef^®^ in the patient chart and tell the recovery room nurse that the patient received Zynrelef^®^.

According to the acute pain service standards, the blocks performed included a Quadratum Lumborum approach (QL) or a Para Vertebral Block (PVB) (unilateral or bilateral open bilateral) for abdominal surgery and an adductor canal block for TKA. All blocks were performed in the same Day of Admission Surgery (DAS) under continuous monitoring of vital signs (heart rate, blood pressure, and oxygen saturation) and using an ultrasound-guided technique. After conducting a “time out,” each patient received intravenous sedation (midazolam 0 mg-2 mg and fentanyl 0 μg-100 μg), and injection of local anesthesia (lidocaine 1% 0 ml-10 ml). Among them lidocaine used for local anesthesia was left at the discretion of the anesthesiologist performing the block. After proper placement of the block needle, 20 ml of either 0.325% bupivacaine or ropivacaine 0.5% was slowly injected after negative aspiration for blood.

Surgery was performed under either general anesthesia (abdominal and foot and ankle surgery) or spinal (TKA). At the end of surgery, the patients were transferred to the recovery room. According to our standard operating procedures, each patient receiving local anesthetic solutions was monitored (vital signs, oxygen saturation, level of consciousness, etc.) for symptoms of LAST (Local Systemic Anesthetic Toxicity). LAST resulted in the administration of intralipid infusion according to our pre-established standard protocol [9,10].

Patient medical history, including age, weight, height, medical history, preoperative medications, type of surgery and its duration, length of hospital stay, type of nerve block performed (location of the block, amount of local anesthetic received), pain (0=no pain to 10=worst possible pain), and opioid consumption until discharge (Oral Opioid Equivalent (OME) in mg) were recorded. The percentage of patients who received an opioid prescription at discharge from the hospital and the percentage of patients who asked for a refill of their prescription were also recorded using the Prescription Drug Monitoring Program (PDMP).

The safety of combination of the use of Zynrelef^®^ and nerve blocks (combination) compared to the use of Zynrelef^®^ alone. Since no LAST related symptoms were recorded in previous clinical studies based on the use of Zynrelef^®^, the safety of the combination was defined as no LAST related symptoms with the combination. Other analysis included a comparison between the group receiving the combination and the group receiving only Zynrelef^®^ with a specific focus on the number of patients who filled their opioids prescription at discharge and those requiring opioid refilled within 30 days following discharge. Differences between Pain scores using a verbal scale (0=no pain and 10=worst possible pain), total consumed OMEs were analyzed. Baseline demographic characteristics were computed. Continuous variables were described by means, Standard Deviations (SD), and categorical variables by counts and percentages. Differences in continuous distributions were tested using the Kruskal-Wallis tests and differences in categorical variables were tested using Fisher’s exact tests. Continuous distributions were visualized using histograms. Missing data was removed from any denominators and any statistical testing. Data are presented as mean ± Standard Deviation (SD).

## Results

Table 1 shows overall patient demographics and pre- and postoperative characteristics. One hundred eighty-four patients received Zynrelef^®^. Qualifying surgeries included unilateral and bilateral inguinal hernia repair (60), ventral or umbilical hernia repair (42), nephrectomy (24), open prostatectomy (28), cystectomy (6), other abdominal surgeries (12), total knee replacement (3), and foot and ankle surgeries (9). Blocks were performed in 159 patients. These blocks included 114 bilateral blocks (113 QL and 2 PVB; 99 performed with bupivacaine and 16 with ropivacaine), 45 unilateral blocks (4 PVB, 37 QL, and 3 adductor canal). Thirty-one blocks were performed with bupivacaine and 13 with ropivacaine. Twenty-five patients did not receive a block. Table 2 shows the numbers and types of blocks performed in patients who received Zynrelef^®^.

Table 3 shows the ambulatory patient demographics and pre- and postoperative characteristics. A total of 95 patients were discharged on the day of surgery, including 75 patients in group 1 (nerve block and Zynrelef^®^) and 20 in group 2 (Zynrelef^®^). Pain and opioid consumption (OME) prior to discharge were similar in both groups (1.9 ± 2.30 *vs.* 1.65 ± 1.87; p=0.467 and 9.75 ± 12.74 mg *vs.* 10.32 ± 12.92 mg; p=0.861, respectively). The number of patients who were discharged without an opioid prescription was 45% in group 1 *vs.* 40% in group 2 (p=0.617). Among the patients who were discharged with a prescription, 71% more patients did not fill their prescriptions in group 1 *vs.* group 2 (28% in group 1 *vs.* 8 % in group 2; p=0.574).

Several deviations from the initial monitoring protocol were recorded. The most frequent deviation was related to the lack of posting the request for Zynrelef^®^ 24 hours prior to surgery (only 4% of cases *vs.* 96% posted on the day of surgery *via* a formal request for Zynrelef^®^ (72%) and in 24% *via* a direct communication between the surgeon and the pharmacist, which bypassed the acute pain service. In this group, two QL perineural catheters and orders for postoperative perineural infusion of local anesthetics were placed prior to patient transfer to the operating room. In another case, an order of postoperative IV lidocaine was placed. The infusion of local anesthetics was prepared but not started in two cases (one perineural and one IV) and in the third case, the QL catheter was infused with lidocaine 0.25% at a rate of 5 ml/hr for 7.5 hours. No patients developed LAST. Three patients undergoing open prostatectomy developed a skin infection. Although, Zynrelef^®^ was administered for open prostatectomy between January 12, 2022 and November 29, 2022, one case of skin infection occurred in May, one in June, and one in July of 2022. Furthermore, no skin infections were observed for any other type of surgeries for which Zynrelef^®^ was used. Finally, the use of Zynrelef^®^ was reported by an orthopedic surgeon to not be effective in one case and be associated with excessive bleeding in another case.

## Discussion

Our data suggest that the combination of Zynrelef^®^ and single blocks performed preoperatively is safe. This conclusion is based on the administration of 115 bilateral blocks and 150 mg of bupivacaine in 91 patients or 200 mg of ropivacaine in 20 patients and the administration of unilateral blocks with 75 mg of bupivacaine in 32 patients or 100 mg of ropivacaine in 16 patients. The majority of these blocks were QL blocks. In this study, PVBs were performed only in six patients (four unilateral and two bilateral) and adductor canal blocks were only performed in three patients. However, it is important to recognize that prior to the introduction of Zynrelef^®^, the safety of the combination of a slow-release formulation over 72 hours and the injection of plain bupivacaine in similar doses had been established for Transversus Abdominis Plane block (TAP), interscalene, femoral, and sciatic blocks [11].

When using a long lasting local anesthetic solution, it is clear that there is no indication for the use of either a continuous nerve block or an intravenous infusion of lidocaine for pain, since the goal of both approaches is to deliver local anesthetics over a period of 24 hours-72 hours following surgery. In our study, one patient received Zynrelef^®^ and an infusion of bupivacaine 0.125% at 5 ml/hr for less than 12 hrs. The patient did not present any evidence of LAST. However, in the absence of additional studies, our group’s recommendation remains to not combine continuous nerve blocks or IV lidocaine infusion and Zynrelef^®^.

As an anti-inflammatory, meloxicam has been reported to induce bleeding. Although, in our series, the orthopedic surgeons reported an excessive bleeding in one patient receiving Zynrelef^®^, it is unlikely that the excessive bleeding was related to meloxicam because the dose of meloxicam in Zynrelef^®^ is very low, and more importantly, the patient’s medical history included a similar complication following her prior primary TKA.

Persistent use of opioids following surgery has been found to be a risk factor for developing opioid use disorder and the associated risk of opioid overdose. Our data suggests that the combination of single nerve blocks and Zynrelef^®^ represents a more effective approach to reduce postoperative dependence on opioids after discharge compared to the use of Zynrelef^®^ alone. Randomized studies have reported that Zynrelef^®^ is associated with an Opioid-Sparing properties [12].

## Conclusion

The study had several limitations. First, it was an observational study and not a randomized study. A randomized study comparing Zynrelef^®^ *vs.* single nerve block plus Zynrelef^®^ would be of interest, especially to confirm the efficacy of the combination. Second, only a limited number of types of nerve blocks were performed. Certainly, the ability to document the safety of the use of Zynrelef^®^ with additional types of blocks would be of interest, as it has been established that local re-absorption of local anesthetics varies with block type. This study provides evidence that the combination of a single nerve block unilateral or bilateral performed prior to surgery with 20 ml of either bupivacaine 0.325% or ropivacaine 0.5% and the application use of Zynrelef^®^ at the end of surgery is safe within the FDA approved indications. Because this study focused mostly on Ql blocks, additional studies are required to confirm the safety of the combination of other types of single blocks and Zynrelef^®^.

## Figures and Tables

**Table 1:** Demographics, preoperative and postoperative characteristics are listed according to whether the subjects received no block, a unilateral block or a bilateral block.

Mean	Total n=184	No block n=25 (14%)	Unilateral block n=45 (24%)	Bilateral block n=114 (62%)
**Age (years)**				
Mean (SD)	63.43 (14.84)	57.6 (17.7)	70.0 (11.9)	62.1 (14.4)
**Height (cm)**				
Mean (SD)	174.52 (10.63)	168.3 (10.6)	177.7 (8.2)	174.6 (11)
**Weight (kg)**				
Mean (SD)	86.01 (17.93)	77.6 (21.1)	83 (16.6)	89 (17.1)
**BMI**				
Mean (SD)	28.18 (5.66)	27 (6.3)	26.5 (5.1)	29.1 (5.6)
**Gender**				
Female % (n)	26.6 (49)	56 (14)	8.9 (4)	27.2 (31)
Male % (n)	73.4 (135)	44 (11)	91.1 (41)	72.8 (83)
**Race**				
African American % (n)	9.2 (17)	4 (1)	4.4 (2)	12.3 (14)
American Indian % (n)	0.5 (1)	0 (0)	0 (0)	0.9 (1)
Asian % (n)	1.1 (2)	4 (1)	0 (0)	0.9 (1)
Declined % (n)	1.1 (2)	4 (1)	2.2 (1)	0 (0)
White % (n)	87.5 (161)	88 (22)	93.3 (42)	85.1 (97)
**Hispanic origin**				
White - Hispanic % (n)	0.5 (1)	0 (0)	0 (0)	0.9 (1)
**History of anxiety**				
No % (n)	62.5 (115)	56 (14)	57.8 (26)	65.8 (75)
Yes % (n)	37.5 (69)	44 (11)	42.2 (19)	34.2 (39)
**History of depression**				
No % (n)	72.8 (134)	60 (15)	73.3 (33)	75.4 (86)
Yes % (n)	27.2 (50)	40 (10)	26.7 (12)	24.6 (28)
**History of chronic pain**				
No % (n)	72.8 (134)	64 (16)	66.7 (30)	77.2 (88)
Yes % (n)	27.2 (50)	36 (9)	33.3 (15)	22.8 (26)
**Preop use of opioids**				
No % (n)	83.7 (154)	72 (18)	84.4 (38)	86 (98)
Yes % (n)	16.3 (30)	28 (7)	15.6 (7)	14 (16)
**PACU pain score prior to opioids**				
Mean (SD)	4.73 (3.56)	4 (3.6)	4 (3.3)	5.2 (3.6)
**PACU pain score after opioids**				
Mean (SD)	3.42 (3.01)	2.4 (2.4)	2.2 (2.4)	4.1 (3.2)
**Discharge pain score**				
Mean (SD)	2.72 (2.69)	1.8 (2)	2 (2.2)	3.2 (2.9)
**Total OME (mg)**				
Mean (SD)	51.87 (116.36)	63.9 (246.5)	20.9 (38.2)	61.5 (89.2)
**Opioid prescription at discharge**				
No % (n)	41.3 (76)	40 (10)	46.7 (21)	39.5 (45)
Yes % (n)	58.7 (108)	60 (15)	53.3 (24)	60.5 (69)
**Opioid prescription filled at discharge**				
No % (n)	17.6 (19)	6.7 (1)	16.7 (4)	20.3 (14)
Yes % (n)	82.4 (89)	93.3 (14)	83.3 (20)	79.7 (55)
**Opioid refill w/in 30 days after discharge**				
No % (n)	39.7 (73)	40 (10)	44.4 (20)	37.7 (43)
Yes % (n)	60.3 (111)	60 (15)	55.6 (25)	62.3 (71)

**Table 2:** Types of surgeries performed on patients who received Zynrelef^®^ and the type of nerve block (if any) that they received.

Type	Total (n=184)	No block (n=25)	Block (n=159)	Unilateral block (n=45)	Bilateral block (n=114)
Inguinal hernia repair	60 (20)*	9	51 (20)*	37 (13)*	14 (7)*
Ventral and/or umbilical hernia repair	42 (5)*	4	38 (5)*	0	38 (5)*
	(1)^§^		(1)^§^		(1)^§^
Nephrectomy	24 (3)^§^	1	23 (3)^§^	4 (4) ^§^	19 (1)^§^
Prostatectomy	28 (1*)	0	28 (1)*	0	28 (1)*
Cystectomy	6 (1)^§^	0	6 (1) ^§^	0	6 (1) ^§^
TKA	3	0	3	3	0
Foot and ankle surgery	9	9	0	0	0
Other	12 (4)*	2	10 (4)*	0	10 (4)*

Note:

* number of blocks performed with ropivacaine;

§ number of PVBs.

**Table 3:** Demographics of patients undergoing ambulatory surgery receiving Zynrelef^®^ patients and who received a block or no block. This table also includes the distribution of patients according to race, history of anxiety, depression, chronic pain and preoperative use of opioids, pain on arrival in PACU, pain 1 hour after the administration of opioid in PACU, total opioid administration prior to discharge, pain at discharge, patients filling their opioid prescription at discharge and patients requesting another opioid prescription after discharge.

Mean	Total (n=95)	Nerve block (n=75)	No nerve block (n=20)
**Age (years)**			
Mean (SD)	61.37 (16.35)	63.7 (15.7)	52.8 (16.4)
**Height (cm)**			
Mean (SD)	175.09 (11.41)	176.8 (10.8)	168.6 (11.5)
**Weight (kg)**			
Mean (SD)	84.55 (17.92)	86 (16.3)	79 (22.5)
**BMI**			
Mean (SD)	27.23 (5.24)	27.2 (4.8)	27.2 (6.8)
**Gender**			
Female % (n)	20 (19)	9.3 (7)	60 (12)
Male % (n)	80 (76)	90.7 (68)	40 (8)
**Race**			
African American % (n)	9.5 (9)	10.7 (8)	5 (1)
American Indian % (n)	1.1 (1)	1.3 (1)	0 (0)
Asian % (n)	2.1 (2)	1.3 (1)	5 (1)
Declined % (n)	2.1 (2)	1.3 (1)	5 (1)
White % (n)	85.3 (81)	85.3 (64)	85 (17)
**Hispanic origin**			
White–Hispanic % (n)	0 (0)	0 (0)	0 (0)
**History of anxiety**			
No % (n)	62.1 (59)	66.7 (50)	45 (9)
Yes % (n)	37.9 (36)	33.3 (25)	55 (11)
**History of depression**			
No % (n)	72.6 (69)	78.7 (59)	50 (10)
Yes % (n)	27.4 (26)	21.3 (16)	50 (10)
**History of chronic pain**			
No % (n)	73.7 (70)	76 (57)	65 (13)
Yes % (n)	26.3 (25)	24 (18)	35 (7)
**Preop use of opioids**			
No % (n)	86.3 (82)	90.7 (68)	70 (14)
Yes % (n)	13.7 (13)	9.3 (7)	30 (6)
**PACU pain score prior to opioids**			
Mean (SD)	4.05 (3.43)	4.1 (3.4)	3.8 (3.6)
**PACU pain score after opioids**			
Mean (SD)	2.44 (2.53)	2.5 (2.6)	2.2 (2.4)
**Total OME (mg)**			
Mean (SD)	9.77 (12.2)	9.5 (12)	10.7 (13.2)
**Discharge pain score**			
Mean (SD)	1.85 (2.19)	2 (2.3)	1.4 (1.8)
**Opioid prescription at discharge**			
No % (n)	47.4 (45)	49.3 (37)	40 (8)
Yes % (n)	52.6 (50)	50.7 (38)	60 (12)
**Opioid prescription filled at discharge**			
No % (n)	57.9 (55)	61.3 (46)	45 (9)
Yes % (n)	42.1 (40)	38.7 (29)	55 (11)
**Opioid refill w/in 30 days after discharge**			
No % (n)	47.4 (45)	49.3 (37)	40 (8)
Yes % (n)	52.6 (50)	50.7 (38)	60 (12)
